# Changes in bone marrow and peripheral blood lymphocyte subset findings with onset of hepatitis-associated aplastic anemia

**DOI:** 10.1097/MD.0000000000028953

**Published:** 2022-02-25

**Authors:** Toshihiko Kakiuchi, Katsuhide Eguchi, Daisuke Koga, Hiroi Eguchi, Masanori Nishi, Motoshi Sonoda, Masataka Ishimura, Muneaki Matsuo

**Affiliations:** a Department of Pediatrics, Faculty of Medicine, Saga University, Saga, Japan; b Department of Pediatrics, Faculty of Medicine, Kyushu University Hospital, Fukuoka, Japan.

**Keywords:** CD4^+^ T cells, CD8+ T cells, cytomegalovirus, hepatitis-associated aplastic anemia, natural killer cells, peripheral blood lymphocyte subset

## Abstract

**Rationale:**

Hepatitis-associated aplastic anemia (HAAA) is a rare illness that results in bone marrow failure following hepatitis development. The etiological agent remains unknown in most HAAA cases. However, clinical features of the disease and immunotherapy response indicate that immune-mediated factors play a central role in the pathogenesis of HAAA. Activation of cytotoxic T cells and increase in CD8 cells could exert cytotoxic effects on the myelopoietic cells in the bone marrow.

**Patient concerns:**

A 15-month-old boy was brought to our hospital with complaints of generalized petechiae and purpura observed a week prior to hospitalization. His liver was palpated 3 cm below the costal margin, platelet count was 0 × 10^4^/μL, and alanine aminotransferase level was 1346 IU/L. A blood test indicated cytomegalovirus infection, and 3 bone marrow examinations revealed progressive HAAA. As the disease progressed to the 3^rd^, 6^th^, and 9^th^ week after onset, CD4^+^ T cells were markedly decreased, CD8^+^ T cells were markedly increased, and the CD4/CD8 ratio was significantly decreased. The number of B cells and natural killer cells decreased with time, eventually reaching 0.0%.

**Diagnosis:**

HAAA.

**Interventions:**

Rabbit antithymocyte globulin and eltrombopag olamine (a thrombopoietin receptor agonist) were administered.

**Outcomes:**

The patient's platelet count returned to normal, and bone marrow transplantation was avoided. The peripheral blood lymphocytes (PBLs) improved as the patient's general condition recovered.

**Lessons:**

This case demonstrates that HAAA induced by cytomegalovirus infection features decreasing CD4^+^ and increasing CD8^+^ PBLs as the bone marrow hypoplasia progresses. The PBLs return to their normal levels with the recovery from the disease. Our case findings thus support the involvement of immunological abnormality in HAAA.

## 1 Introduction

Hepatitis-associated aplastic anemia (HAAA) is a well-recognized clinical syndrome in which bone marrow failure follows the development of hepatitis.^[1–6]^ HAAA was first reported in 1955^[1]^ and is considered a major form of aplastic anemia, with severe leukopenia occurring 2 to 3 months after acute hepatitis.^[2,4]^ HAAA has been defined as a variant of aplastic anemia (AA) in which pancytopenia occurs concurrently or within 6 months of increase in serum alanine aminotransferase (ALT) levels to more than 5 times the upper limit of normal.^[3,7]^ The diagnosis is based on National Institutes of Health reports.^[2]^ Among 3916 patients with AA reported to the European registry from 1990 to 2007, HAAA accounted for 1% to 5% of all cases.^[8]^ The incidence is high in places where hepatitis is prevalent, mostly in Asian countries^[9,10]^ and in areas of low socioeconomic status.^[4,11]^ The etiological agent remains unknown in most cases of HAAA.^[2,5,11]^

In some studies of patients with HAAA, several immunological abnormalities that responded favorably to immunosuppressive treatment have been described.^[2,5,12,13]^ These findings suggest that the underlying pathological mechanism in HAAA may be immune mediated.

Here, we present a pediatric case of AA caused by cytomegalovirus (CMV) hepatitis. To the best of our knowledge, this is the first case in which changes in bone marrow findings as well as the time course of the peripheral blood lymphocytes (PBLs) subset were observed simultaneously with HAAA progression. This case is important because it describes the time course of the immunological mechanism in the development of HAAA and recovery from it.

## 2 Case report

A previously healthy 15-month-old boy was brought to our hospital with complaints of generalized petechiae and purpura observed a week prior to hospitalization. He was born without any problems and had not developed any health problems. His parents too did not have any health problems. He had no siblings, and he was on track with the national vaccination program in Japan. On his initial visit to our hospital, the patient's vital signs were almost normal. His body temperature was 37.1°C, blood pressure was 98/58 mm Hg, heart rate was 120 beats per minute, and saturation of percutaneous oxygen was 98% without supplemental oxygen. His height was 76.8 cm (standard deviation: −0.37 cm), and his weight was 9.4 kg (standard deviation: −0.52 kg). A physical examination revealed spotted hemorrhage and small purpuras scattered throughout the body. Frequent mucosal bleeding in the mouth was noted. The liver was palpated 3 cm below the costal margin but not the spleen.

His blood test results are shown in Table 1, and the most significant findings were as follows: White blood cell count was 1600/μL (normal range: 7000-15,000), hemoglobin level was 9.3 g/dL (normal range: 13.7-16.8), and platelet count was 0 cells/μL. Moreover, his laboratory test findings showed significantly elevated enzyme levels, as follows: aspartic aminotransferase 909 IU/L (normal range: 20-45 IU/L); ALT 1346 IU/L (normal range: 4-24 IU/L); gamma-glutamyl transpeptidase 140 IU/L (normal range: 5-17 IU/L); and lactic acid dehydrogenase 441 IU/L (normal range: 245-427 IU/L). Furthermore, the total bilirubin and albumin levels were 1.2 mg/dL (normal range: 0.3-1.2 mg/dL) and 4.0 g/dL (normal range: 2.5-4.0 mg/dL), respectively. The time of onset of liver damage could not be determined based on medical history and physical findings. Evidences for infection with hepatitis B virus (HBV), hepatitis C virus, Epstein–Barr virus (EBV), and herpes simplex virus were not found. However, CMV immunoglobulin M antibody (cutoff index value: 2.73; normal range: <0.8), immunoglobulin G antibody (16.0 AU/mL; normal range: <6.0 AU/mL), and CMV deoxyribonucleic acid (DNA) (200 copies/10^6^ cells; normal range: <2.0 × 10^1^/10^6^ cells) were positive, which suggested primary infection with CMV. His autoimmunology-related test values (ANA, dsDNA-Ab[antibody], ssDNA-Ab, liver-kidney microsome 1-Ab, and anti-smooth muscle antibody) were all negative. His abdominal ultrasound showed mild thickening of the gallbladder wall and increased brightness around the portal vein (Figure S1, Supplemental Digital Content). Bone marrow examination was performed to determine the cause for reduction in white blood cell and platelet count. Bone marrow smear revealed slight hypoplasia, with a nucleated cell count (NCC) of 80,000/μL. The cells were not atypical, and abnormal cell proliferations were not observed. Several small lymphocytes with a very high nuclear–cytoplasmic ratio were observed, and megakaryocytes were small in number and size. Furthermore, poor platelet adhesion was noted (Figure S2, Supplemental Digital Content). No obvious trilineage dysplasia was observed in peripheral blood and bone marrow smears, and blast cells were not present. The diagnosis at this point was CMV hepatitis and myelosuppression owing to CMV infection with negative AA.

**Table 1:** Patient's blood test and biochemical test results at the first visit.

WBC	1,600	/μL	TP	6.3	g/dL	HBs-Ag	0	IU/mL
Stab	0.5	%	Alb	4	g/dL	HCV-Ab	0	C.O. I
Seg	50.5	%	T-Bil	1.2	mg/dL	CMV-IgM	2.73	C.O. I
Lym	38	%	D-Bil	0.2	mg/dL	CMV-IgG	16	AU/mL
Mo	3.6	%	AST	909	IU/L	CMV-DNA (PCR)	200	copies/10^6^ cells
Eosi	1	%	ALT	1346	IU/L	EBV-IgM	<10	C.O. I
Aty-Ly	0.5	%	LDH	441	IU/L	EBV-IgA	<10	C.O. I
RBC	373 × 10^3^	/μL	GGT	140	IU/L	EBV-IgG	<10	C.O. I
Hb	9.3	g/dL	BUN	12.7	mg/dL	EBNA	<10	C.O. I
MCV	77.2	fL	Cre	0.23	mg/dL	HSV-IgM	<2.0	C.O. I
MCHC	32.3	%	TG	46	mg/dL	HSV-IgG	<2.0	C.O. I
Plt	0	/μL	T-chol	137	mg/dL			
			Na	139	mEq/L	ANA	<40	
IgG	653	mg/dL	K	3.9	mEq/L	dsDNA-Ab	<10	IU/mL
IgA	50	mg/dL	Cl	102	mEq/L	ssDNA-Ab	<6.0	U/mL
IgM	33	mg/dL	CRP	0.03	mg/dL	LKM1-Ab	<17	
IgE	258	IU/mL	Ferritin	34.1	ng/mL	ASMA	<40	

ASMA = anti-smooth muscle antibody, C.O. I = cut-off index, CMV = cytomegalovirus, EBV = Epstein–Barr virus, HCV = hepatitis C virus, IgG = immunoglobulin G, IgM = immunoglobulin M, LKM1-Ab = liver-kidney microsome 1 antibody, PCR = polymerase chain reaction.

**Figure 1:**
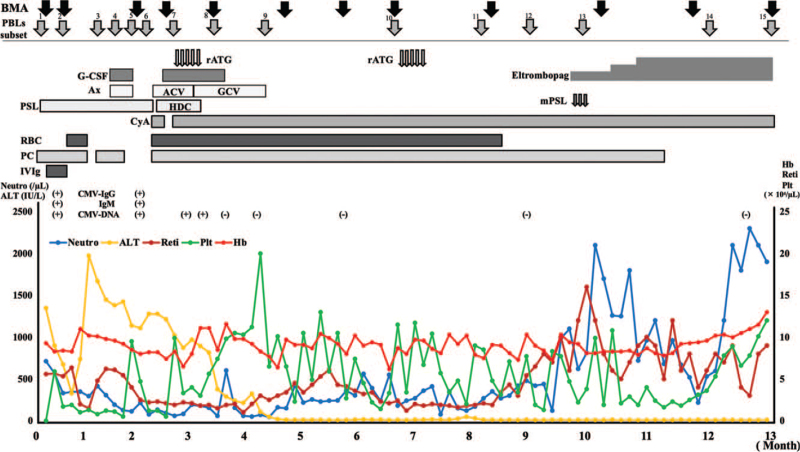
The patient's clinical course. Pancytopenia was refractory to prednisolone, cyclosporine, intravenous immunoglobulin, and blood transfusions. Liver dysfunction improved rapidly with treatment for cytomegalovirus, but pancytopenia did not. For pancytopenia, the first rabbit antithymocyte globulin was shown to be therapeutic; however, the patient did not respond to the second dose and eltrombopag olamine was ultimately effective. During the clinical course, 12 bone marrow aspirations and 15 peripheral blood lymphocytes analyses were performed. ACV = acyclovir, Ax = antibiotic agent, BMA = bone marrow aspiration, CyA = cyclosporin, G-CSF = granulocyte-colony stimulating factor, GCV = ganciclovir, HDC = hydrocortisone, IVIg = intravenous immunoglobulin, mPSL = methylprednisolone, PBLs = peripheral blood lymphocytes, PC = platelet, PSL = prednisolone, rATG = rabbit antithymocyte globulin, RBC = red blood cell, Reti = reticulocyte.

**Figure 2:**
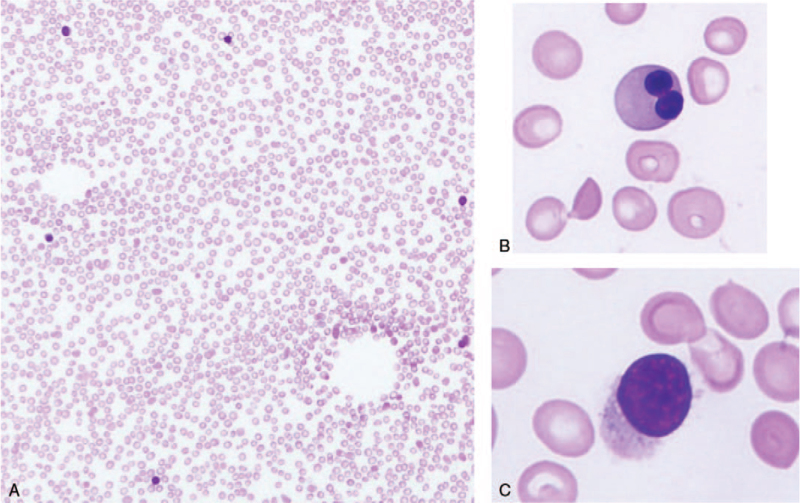
The third bone marrow smear indicating marked hypoplasia and a nucleated cell count of 1000/μL (A). Dysplasia was observed in erythroblasts (B), and megakaryocytes were small (C).

The patient's clinical course is shown in Figure 1. Initially, CMV infection was not treated, given the side effects of the therapeutic agents. Platelet count showed no favorable response after globulin administration and worsened in response to platelet transfusion. Hepatic disorders improved slightly, with the corresponding resolution of hepatomegaly. Two weeks after the first bone marrow examination, a second examination was performed (Figure S3, Supplemental Digital Content). The second bone marrow smear indicated hypoplasia and an NCC of 25,000/μL. The macrophages were conspicuous, and atypical lymphocytes were also present. Similar to the first examination, abnormal cell proliferations were not observed. Moreover, CMV-DNA was not detected in the bone marrow fluid. The subsequent laboratory test findings were as follows: fibrinogen levels were 189.5 mg/dL (95^th^ percentile; normal range: 145-348 mg/dL), triglyceride levels were 88 mg/dL (95^th^ percentile; normal range: 24-173 mg/dL), and ferritin levels were 234 ng/mL (95^th^ percentile; normal range: 8-150 ng/mL). The patient was diagnosed with CMV hepatitis and phagocytosis in the bone marrow due to CMV infection, with no suspicion of AA. He did not meet the diagnostic criteria for hemophagocytotic lymphohistiocytosis (HLH). After the administration of steroids, the number of platelet transfusions decreased; however, the platelet count did not exceed 20,000, and the liver disorder persisted. The third bone marrow examination was performed 8 weeks after the first one (Fig. 2). The bone marrow smear showed marked hypoplasia and an NCC of 1000/μL. The megakaryocytes were small, and dysplasia was observed in erythroblasts. Immunostaining of the bone marrow showed many CD8-positive cells but no CD4-positive cells (Figure S4, Supplemental Digital Content). The thrombopoietin level was markedly elevated (35.8 fmol/mL; normal range, 2.23 ± 0.89 fmol/mL).^[14]^ The patient was diagnosed with HAAA based on the presence of CMV hepatitis and severe AA (absolute neutrophil count: 113/μL; platelet count: 0.6 × 10^4^/μL) due to CMV hepatitis.^[15]^

**Figure 3:**
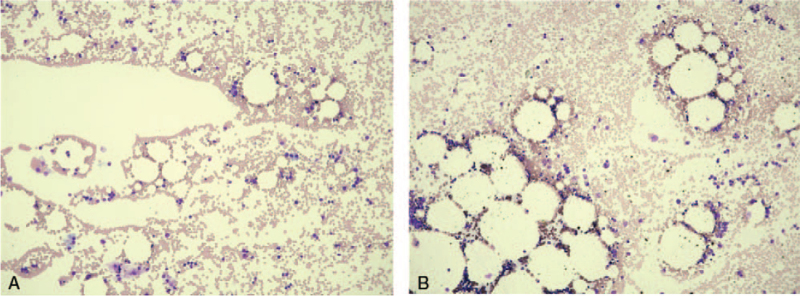
The bone marrow smear performed 4 months after the first administration of rATG showed moderate hypoplasia and an NCC of 18,200/μL. Megakaryocytes were small and did not indicate platelet production (A). After eltrombopag olamine administration, the bone marrow smear showed normal formation and an NCC of 48,000/μL. Megakaryocytes were increased, and dysplasia was not observed (B).

The patient was shifted to another hospital for bone marrow transplantation for HAAA. The liver biopsy findings showed minimal lymphocytic infiltration in the portal areas. Steatosis, cholestasis, and fibrosis were not evident. Cytomegalic inclusion body and anticytomegalovirus antibody reactivity were not evident (Figure S5, Supplemental Digital Content). CMV-DNA quantification value using liver tissue was 7.0 × 10^2^ copy/mL (normal range: <4.0 × 10^1^ copy/mL). Acyclovir and ganciclovir were administered for CMV infection after the hospital transfer, and the CMV-DNA in the blood consequently disappeared. Before bone marrow transplantation for HAAA, rabbit antithymocyte globulin (rATG) was challenged with a cyclosporine combination (trough value: 150-250 ng/mL), and the platelet count recovered to approximately 10.0 × 10^4^/μL. However, 1 month later, the platelet count decreased to ≤50,000/μL, and blood transfusion was required. Up to this point, the bone marrow smear showed hypoplasia. The bone marrow smear performed 4 months after the first administration of rATG showed moderate hypoplasia and an NCC of 18,200/μL. The megakaryocytes were small and did not indicate platelet production (Fig. 3A). The recovery of the blood cell system was not sufficient even after the second administration of rATG; however, bone marrow smear gradually showed the recovery of bone marrow formation. Eventually, eltrombopag olamine (a thrombopoietin receptor agonist) was administered, which returned the platelet count to normal and helped avoid bone marrow transplantation. The bone marrow smear showed normal formation and an NCC of 48,000/μL. The number of megakaryocytes increased, and dysplasia was not observed (Fig. 3B). No obvious trilineage dysplasia or blast cells were observed in peripheral blood or bone marrow smears in any of the bone marrow examinations performed during the course of the disease. During the manifestation of HAAA, the patient did not experience life-threating bleeding episodes or serious infections. Furthermore, there were no findings suggesting the onset of acute leukemia during the clinical course.

Table 2 shows the time course of PBL subset results. As the disease progressed to the 3^rd^, 6^th^, and 9^th^ week after the onset, CD4^+^ T cells were markedly decreased, CD8^+^ T cells were markedly increased, and the CD4/CD8 ratio was significantly lowered. B cells and natural killer (NK) cells decreased over time and eventually reached 0.0%. T cell receptor recombination circles, kappa-chain recombination excision circles, and severe combined immunodeficiency mutation screening test showed no abnormalities suggestive of acquired immunodeficiency. Furthermore, human immunodeficiency virus antigen antibody test was negative. As the treatment progressed, the significantly decreased CD4^+^ T cells returned to their normal values and the significantly increased CD8^+^ T cells decreased to their normal values. Consequently, the CD4/CD8 ratio was normalized. B cells and NK cells showed an upward trend over time. This posttreatment result was the opposite of what was noted during the progression of HAAA.

**Table 2:** Time course of peripheral blood lymphocyte subsets.

	Day 6	Day 19	Day 37	Day 45	Day 55	Day 61	Day 89	Day 99	Day 123	Day 198	Day 224	Day 280	Day 311	Day 341	Day 400	
	^∗^1	2	3	4	5	6	7	8	9	10	11	12	13	14	15	
T cell	552	431	596	554	589	705	747	459	370	301	539	889	974	1,295	1,952	/μL
CD4^+^	49	37	26	21	19	22	71	69	39	186	286	480	661	846	1,196	/μL
CD8^+^	367	320	489	621	578	613	562	602	274	167	421	455	497	680	983	/μL
CD4^+^/CD8^+^ ratio	0.13	0.12	0.05	0.03	0.03	0.03	0.12	0.11	0.14	1.11	0.68	1.05	1.33	1.24	1.22	
B cell	144	147	1	0	0	0	0	11	31	9	45	66	71	163	221	/μL
NK cell	21	10	1	0	0	0	0	24	43	42	105	101	97	356	53	/μL

Day, day from admission. NK cell = natural killer cell.

∗ Numbers, see Figure 1.

At the most recent evaluation 4 years after commencing eltrombopag olamine administration, the patient was healthy without the need for medication and was being carefully monitored for the development of leukemia.

## 3 Discussion

The patient's clinical course reveals 2 important points. First, we were able to follow the progression of HAAA and recovery from the disease with the aid of bone marrow and PBL subset findings. Hence, we were able to observe the decrease in CD4^+^ and increase in CD8^+^ PBLs as the bone marrow hypoplasia progressed. Conversely, the findings normalized with the recovery from HAAA. Second, this HAAA case was most probably induced by CMV infection as per the serum CMV antibody and polymerase chain reaction findings using peripheral blood samples.

As in the present case, when anemia is present at the onset along with severe thrombocytopenia, diseases other than HAAA, such as infantile giant cell hepatitis (GCH) with autoimmune hemolytic anemia, must be differentiated. GCH is characterized by large and multinucleated hepatocytes in the context of liver inflammation. Infantile GCH is typically associated with autoimmune hemolytic anemia.^[16,17]^ In our case, the lack of hemolytic anemia in the initial laboratory findings was sufficient to eliminate GCH. In addition, the bone marrow findings ruled out other diagnoses, such as hemophagocytic syndrome, megaloblastic anemia, bone marrow infiltration of malignancy, myelodysplastic syndrome, acute leukemia, and thymoma.^[18,19]^ Myelodysplastic syndrome was excluded based on the absence of precursor cells, blood cell atypia, and chromosomal abnormalities. Flow cytometry did not detect glycosylphosphatidylinositol-anchored protein-deficient blood cells; therefore, paroxysmal nocturnal hemoglobinuria was ruled out. Chromosomal breakage in PBL was not evaluated; however, physical findings and skin pigmentation unique to Fanconi anemia, such as congenital anemia, were observed.

The clinical features of the disease and its immunotherapy response indicate that immune-mediated factors play a central role in the pathogenesis of HAAA.^[9]^ Recent studies have demonstrated the simultaneous expansion of a liver-infiltrating cytotoxic T lymphocyte clone with the development of HAAA.^[20]^ Moreover, CD8^+^ Kupffer cells might be important mediators of HAAA.^[5]^ Brown et al^[2]^ reported that patients with HAAA have a decreased CD4/CD8 ratio in peripheral blood, which is associated with the activation of cytotoxic T cells and an increase in the proportion of HLA-DR-positive CD8 cells. It has been reported that the phenomenon in HAAA appears more clearly than in idiopathic AA.^[21]^ Kagan et al^[22]^ demonstrated in vitro that in AA, activated CD8-positive lymphocytes could be cytotoxic to the myelopoietic cells in the bone marrow. In our case, PBL subset analysis was performed during the gradual decrease in NCC in the bone marrow, and eventually the diagnosis of AA was reached. The PBL subset analysis demonstrated a decrease in the number of CD4^+^ cells and an increase in the number of CD8^+^ cells, which decreased the CD4^+^/CD8^+^ ratio. To the best of our knowledge, this is the first pediatric case describing consecutive bone marrow findings leading to HAAA and a decrease in the CD4^+^/CD8^+^ ratio using PBL analysis. Our findings support that the increase in CD8^+^ cells is responsible for the bone marrow hypoplasia in HAAA. Furthermore, solid evidence could be obtained because the changes in the lymphocyte subset were also monitored during the recovery from HAAA with the treatment. Several other studies have demonstrated that activated cytotoxic lymphocytes produce increased amounts of interferon (IFN)-γ in the bone marrow of patients with AA.^[12,23,24]^ IFN-γ is a marrow-suppressing cytokine that could induce bone marrow inhibition.^[23]^ Hepatic inflammation in HAAA is linked to analogous findings.^[25]^ In our case, unfortunately, we could not prove this theory because we could not measure inflammatory cytokines over time.

The PBL subset analysis showed that the proportion of NK cells decreased with the onset of HAAA and finally reached 0%. Similar findings have also been reported in idiopathic AA.^[26]^ Gascon et al^[27]^ documented that deficient NK cell activity was an intrinsic feature of AA and that the recovery of NK cell activity was related to hematopoietic recovery. The results from the present study support their theory. The immunological significance of NK cell activity depletion in HAAA, similar to AA, requires further research.

The association between AA and HLH has been debated, particularly the aspect that HLH may be a cause for secondary AA.^[28,29]^ HAAA cases featuring bone marrow infiltration of macrophages and hemophagocytosis have been reported.^[30]^ Because HLH is known to cause aplastic bone marrow if left untreated for a prolonged duration, HAAA may thus be associated with HLH. AA-associated HLH has rarely been documented, and problems in the diagnostic procedure have been discussed.^[31]^ In the present case, the second bone marrow examination showed conspicuous macrophages. Although this observation does not satisfy the HLH diagnostic criteria,^[32]^ changes in cytokine levels associated with hepatitis resulted in an HLH-like pathology after acute hepatitis. Moreover, increased CTL, IFN-γ, and tumor necrosis factor-α might have led to the onset of AA. On the other hand, Brisse et al^[33]^ reported that CD8^+^ T cells constitute a defensive factor in virus-associated secondary HLH. In the present case, it cannot be denied that the increase in CD8^+^ cells with the progress to HAAA might have exerted a protective effect against HLH associated with CMV. We could not clearly explain the cause for the increase in macrophages in the second bone marrow examination. Further accumulation and evaluation of HAAA cases with HLH may lead to an adaptive expansion of HAAA etiology.

We also hypothesize that this HAAA case was induced by CMV infection. AA cases associated with hepatitis A virus (HAV), HBV, hepatitis G virus, human parvovirus B19, EBV, and transfusion-transmitted virus or echovirus infections have been reported.^[25]^ However, the etiological agent remains unknown in most AA cases.^[2,5,13,25]^ In a study in Japan, 61 HAAA cases were observed in 525 children with acquired AA.^[33]^ Viral studies of 49 of the 61 cases revealed single patients infected with HAV, hepatitis C virus, EBV, or CMV. In 214 HAAA cases retrieved from the European registry between 1990 and 2007, no causative virus for hepatitis was identified in 94% of the patients with HAAA, whereas it was a hepatitis virus in 15 cases—HBV in 9 cases and HAV in 6 cases.^[8]^ In our case, CMV infection was strongly inferred from the serological findings of CMV and CMV-DNA in blood and liver tissue. The gold-standard assay for CMV is CMV–polymerase chain reaction, which has a reported sensitivity of 85% and specificity of 95%.^[34,35]^ In the present case, although cytomegalic inclusion bodies were not detected in the liver, the CMV-DNA was detected in blood and liver tissue. In addition, the CMV-DNA disappeared with the treatment and the liver dysfunction (ALT value) was reversed, which provides strong evidence that hepatitis was caused by CMV. As identifying the virus causing hepatitis in HAAA may be very difficult, large epidemiological studies that consider regional and racial differences are required to comprehend the relationship between the etiology of hepatitis and HAAA.^[9]^

The present case report has several limitations, including the lack of chromosomal breakage evaluation, the lack of information on changes in inflammatory cytokine levels over time, and the lack of insight into the immunological significance of the depletion of NK cell activity.

Our case report describes the process of bone marrow hypoplasia progression based on consecutive test findings detailing the decrease in CD4^+^ and increase in CD8^+^ PBLs in a pediatric patient. These findings provide evidence supporting the role of immunological abnormalities in the onset of HAAA. Furthermore, our findings show that HAAA may be induced by CMV.

## Acknowledgments

The authors thank the patient's family for providing consent and granting permission to draft and publish this case report.

## Author contributions

**Conceptualization**: Toshihiko Kakiuchi

**Data curation**: Toshihiko Kakiuchi, Katsuhide Eguchi, Daisuke Koga, Hiroi Eguchi, Masanori Nishi, Motohiro Sonoda, and Masataka Ishihara

**Formal analysis**: Toshihiko Kakiuchi

**Investigation**: Toshihiko Kakiuchi, Katsuhide Eguchi, Daisuke Koga, Hiroi Eguchi, Masanori Nishi, Motohiro Sonoda, and Masataka Ishihara

**Methodology**: Toshihiko Kakiuchi

**Project administration**: Muneaki Matsuo

**Supervision**: Muneaki Matsuo

**Validation**: Toshihiko Kakiuchi

**Writing – original draft**: Toshihiko Kakiuchi and Daisuke Koga

**Writing – reviewing & editing**: Muneaki Matsuo

## Supplementary Material

Supplemental Digital Content

## Supplementary Material

Supplemental Digital Content

## Supplementary Material

Supplemental Digital Content

## Supplementary Material

Supplemental Digital Content

## Supplementary Material

Supplemental Digital Content
