# Supplementary material for: Changes in bone marrow and peripheral blood lymphocyte subset findings with onset of hepatitis-associated aplastic anemia
Source: Medicine (Baltimore). 2022 Feb 25;101(8):e28953. doi: 10.1097/MD.0000000000028953 (PMC8878616; doi:10.1097/MD.0000000000028953)

Figure S2. On admission, the bone marrow smear images were slightly hypoplastic and the nucleated cell count was 80,000/µL. Atypical cells and abnormal cell proliferations were not observed (A). Several small lymphocytes with a very high nuclear–cytoplasmic ratio were observed (B). Megakaryocytes were small in number and size, and poor platelet adhesion was noted (C).


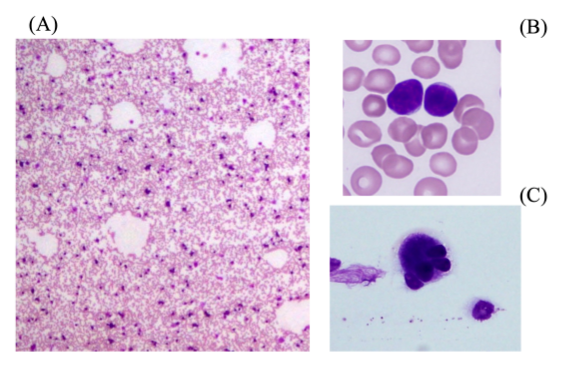

Supplement: Supplemental Digital Content [file medi-101-e28953-s002.docx]
