# Supplementary material for: Changes in bone marrow and peripheral blood lymphocyte subset findings with onset of hepatitis-associated aplastic anemia
Source: Medicine (Baltimore). 2022 Feb 25;101(8):e28953. doi: 10.1097/MD.0000000000028953 (PMC8878616; doi:10.1097/MD.0000000000028953)

Figure S3. Two weeks after the first bone marrow examination, a second examination was performed. The bone marrow smear showed hypoplasia and a nucleated cell count of 25,000/µL (A). Atypical lymphocytes were also present (B), and macrophages were conspicuous (C). Similar to the first bone marrow examination, abnormal cell proliferations were not observed.


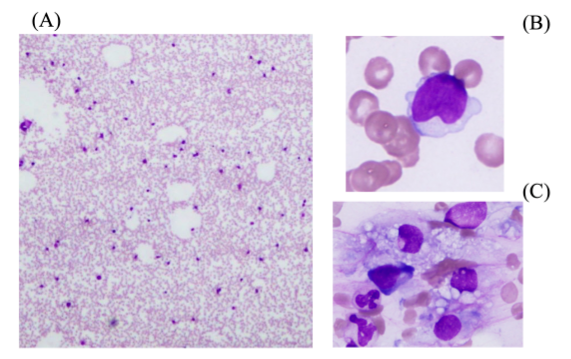

Supplement: Supplemental Digital Content [file medi-101-e28953-s003.docx]
