# Supplementary material for: Changes in bone marrow and peripheral blood lymphocyte subset findings with onset of hepatitis-associated aplastic anemia
Source: Medicine (Baltimore). 2022 Feb 25;101(8):e28953. doi: 10.1097/MD.0000000000028953 (PMC8878616; doi:10.1097/MD.0000000000028953)

Figure S5. Liver biopsy findings showing minimal lymphocytic infiltration in the portal areas. Steatosis, cholestasis, and fibrosis were not evident (A–C). Cytomegalic inclusion body and anti-CMV antibody reaction were not detected.


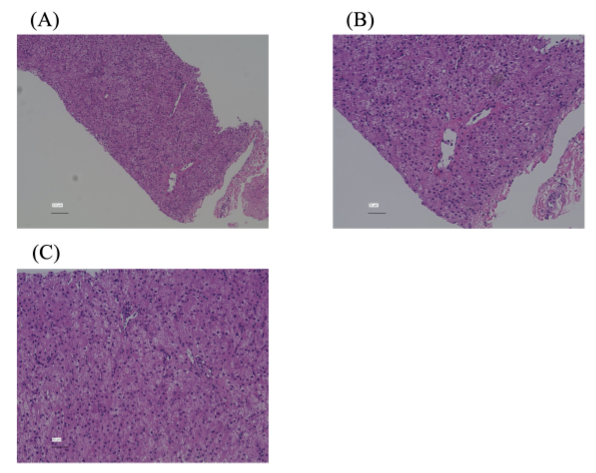

Supplement: Supplemental Digital Content [file medi-101-e28953-s005.docx]
